# Folia Therapeutica

**Published:** 1907-09

**Authors:** 


### Folia Therapeutica.

THE second issue of *Folia Therapeutica,* a journal devoted wholly to therapeutics, has made its appearance and bears out all the promise made in the first issue. As announced by the publishers it is a "periodical journal relating to modern therapeutics and pharmacology for medical practitioners," edited by A. Baginsky, M.D., professor in the Medical University of Berlin, and J. Snowman, M.D., M.R.C.P., of London.

The first impression one receives on taking up this publication is its quiet dignity and the solidity of its contributions. There is new and interesting material on every page and what is more important and gratifying the articles are short, direct and meaty. The authors have a statement to make and they make it without spraddling over several pages of explanation. A resume of the contents of this number of *Folia Therapeutica* will give an excellent idea of its scope and its value. The leading article is a Berlin letter contributed by Prof. C. A. Ewald, and this is followed by a series of original articles: Fuming inhalations in asthma, Sir James Sawyer; On the course and treatment of arteriosclerosis, Prof. Herman Senator, Berlin; Iodipin in the treatment of tertiary syphilis, G. G. Stopford Taylor, Liverpool; On the treatment of gonorrhea in the male, Arnold Edwards, Manchester; Theory and application of the hyperemia treatment of Bier, Dr. V. Schmieden, Bonn; The treatment of psoriasis, Frank H. Barendt, Liverpool; Dionine in ophthalmic practice, Karl Grossmann, Liverpool; Some views on the hydrotherapeutic treatment of a few constitutional diseases, Prof. L. Brieger, Berlin; abstracts from articles on therapeutics; reviews of books on therapeutics; current literature on therapeutics.

The journal is most excellently edited, it is well printed and the illustrations inserted are of exceptional character. *Folia*

*Therapeutica* is one of the most valuable publications of its kind in the English language and should have a wide circulation in this country. It is published quarterly by John Bale, Sons & Danielsson, 83-91 Great Titchfield street, Oxford streets, W. London, Eng.

---

GOVERNOR HUGHES has rendered the medical profession of the state of New York yeoman service, by his veto of the optometry bill which was passed by the last legislature. For several years previously this bill has been defeated, either in committee or in one house or the other, through the combined action of the legislative committees of the several state medical societies; but this year the legislature yielded to the importunities of the opticians. The governor filed the following memorandum with his veto:

> It is the intent of this bill that the board of examiners in optometry, to be appointed by the Board of Regents, shall be selected from those nominated by the Optical Society. It is also provided that the prescribed course of professional study in schools of optometry shall be had in such schools as maintain a standard satisfactory to the board of examiners. These provisions remove from the jurisdiction of the Board of Regents matters which it is important should be placed in their control. If the practice of optometry is to have the recognition and regulation contemplated by this bill the appointment of examiners should not be limited to those nominated by a particular society, and the determination of the standards of professional schools should be unequivocally left to the proper state authority. This is the policy established by the law enacted this year regulating the practice of medicine, and in my judgment it is unwise in legislation along similar lines to adopt a different principle.
>
> (Signed) CHARLES E. HUGHES.

---

SEVERAL of the larger and stronger old line insurance companies have restored the usual medical examination fee of $5.00, which was lowered two or three years ago in the interest of so-called economy. These organisations evidently have learned that it is no economy to cheapen the services of their medical examiners in this manner. Their action in returning to the former custom is commendable, and it is to be hoped that companies representing other forms of insurance will follow the good example set by these standard companies.

---

"OLD HOME WEEK" is just beginning, as this issue goes out to the mails. Buffalo is full of visitors who sometime claimed residence here, or who are otherwise interested in visiting the city to witness its growth and prosperity, but especially to be present during the festivities incident to a week of holiday display and entertainment. Main street is elaborately decorated and many private residences in other streets and avenues have put on holiday adornment. Labor day leads off with its elaborate parade, and other special days, including the dedication of the McKinley monument, contribute to give variety and interest to the week. There is music by day and fireworks by night. 'Tis well!

---

THE sudden death by drowning of the Rev. Arthur S. Mann, a missionary at Kuling, China, caused a shock to his many friends in this city. He was a son of Dr. Matthew D. Mann, of Buffalo, a graduate of Yale (99) and of the General Theological Seminary at New York. The Rev. Mr. Mann was 28 years old at the time of his death and lost his life in attempting to save his companion and friend, the Rev. Warren D. Seabury, in which he was unsuccessful. The sympathies of this community go out to the afflicted family.

---

"SUNDOWN DOCTORS" according to the Washington correspondent of *The Louisville Courier-Journal,* are an institution peculiar to Washington city. They are an amiable company of medical practitioners who ply their trade only after nightfall. Not that these gentlemen prefer darkness to light if they had their "d'ruthers," nor are their deeds of questionable complexion that look best in the shade. "Sundown doctors" have no "ways that are dark" or "tricks that are vain." They are as open as the day that they may not utilise. If they practise their profession by candle light rather than by the sunshine, that's Uncle Sam's fault, not their own. Sundown doctors begin to get busy only after 4:30 in the afternoon. From 9 to that hour, poor souls, they are holding their noses to the grindstones over government desks. For one must live, don't you know, however soaring one's scientific ambition, and Uncle Sam's wages do come in mighty regular and handy. So that in a pigeon hole is the story of the origin of the struggling fraternity of sundown physicians at the federal capital.

There are thousands of instances. Embryonic young physicians, with their careers yet to carve, secure a clerkship in some of the governmental departments of Washington, in order to keep the pot bubbling while they are getting their medical education after office hours. Their diploma thus laboriously achieved, they hang out their shingles, tenatively holding fast, however, to their government position until securely established professionally. A job in the hand, you know, is worth a whole city directory full of uncaptured patients. Never let go a sure thing till you are sure of a surer.

The foregoing comment on an evil that should not exist in these days of improvement in medical education, shows how the laity views the question. From the professional viewpoint there should be no "sundown" colleges. The old ones at best should be permitted to die out, and no new ones should be chartered. They are a sad commentary on higher educational standards in medicine.

